# Draft Genome Sequence of a Trimethylamine-Producing *Staphylococcus* Isolate from Blood of a Coronary Atherosclerotic Heart Disease Patient

**DOI:** 10.1128/genomeA.01563-17

**Published:** 2018-02-01

**Authors:** Xue Yu, Ying-Qian Kang, Fusui Ji

**Affiliations:** a Department of Cardiovascular Medicine, Beijing Hospital, National Center of Gerontology, Beijing, People’s Republic of China; b Department of Microbiology, Guizhou Medical University, Guiyang, People’s Republic of China

## Abstract

Trimethylamine (TMA), which is produced by various bacteria, is associated with heart disease, but little information about the production of TMA in blood is available. We present here the genome sequence of a multidrug-resistant strain, *Staphylococcus epidermidis* bTMA-013, with a TMA synthesis pathway, which was isolated from the blood of a coronary atherosclerotic heart disease patient.

## GENOME ANNOUNCEMENT

Trimethylamine (TMA) is a microbial metabolite that is produced by various bacteria from dietary quaternary amines, mainly choline and carnitine (1). In the human body, TMA is further oxidized to trimethylamine-*N*-oxide (TMAO), which has been associated with atherosclerosis and severe cardiovascular disease in several independent studies (2). Previous studies have reported various pathogenic Gram-negative strains, but *Staphylococcus* spp. carry genes related to TMA biosynthesis (1). *Staphylococcus epidermidis*, a Gram-positive bacterium, is internationally acknowledged as a multidrug-resistant (MDR) human pathogen (3). Here, we describe the whole-genome sequence of strain bTMA-013, which has a complete TMA synthesis pathway. To our knowledge, this is the first isolate of an MDR *S. epidermidis* strain with multiple copies of the gene cluster related to TMA metabolism.

Strain bTMA-013 was isolated from the blood of a coronary atherosclerotic heart disease patient. Preenrichment was done on tryptic soy broth (Oxoid, United Kingdom), followed by incubation at 35°C for 18 to 24 h. DNA was extracted using a genomic DNA isolation kit (Qiagen, Germany). The concentration of extracted DNA was quantified using a PicoGreen double-stranded DNA (dsDNA) assay kit (Invitrogen, USA), and the presence of contamination was checked by sequencing the 16S rRNA gene using the ABI 3730 DNA sequencing machine (Applied Biosystems, USA). The Illumina HiSeq 2500 platform was used for whole-genome sequencing of this clone, which generated 4,893,928 short-read sequences in pairs of about 300 bp, totaling 611.7 Mb, with a G+C content of 35.28%. *De novo* genome assembly was performed using Velvet version 1.2.10 (4), and further downstream processing was performed. Genome annotation was performed with the Rapid Annotations using Subsystems Technology (RAST) server. Overall, we obtained a genome of 2,605,061 bp for strain bTMA-013, and a total of 390 subsystems were identified by RAST (5). The genome contained 2,467 predicted protein-coding sequences that had at least one hit in the SEED database, 59 tRNAs, and 7 complete rRNAs. *In silico* multilocus sequence typing (MLST) of bTMA-013 was performed with the MLST version 1.8 online server (6) and found that this isolate belongs to sequence type 22.

The major TMA synthesis pathway—including the choline TMA-lyase (*cutC*) and its activator *cutD*, which takes choline as a substrate and a two-component Rieske-type oxygenase/reductase (*cntA/B*) as its key enzymes—has been carefully described (7). In strain bTMA-013, we identified one copy of the *cutC/D* gene cluster with a partial *cutC* gene. Based on analysis of the phylogenetic trees, we found that these genes might be obtained from *Enterobacter* spp. through horizontal gene transfer, implying that bTMA-013 has the ability to convert ammonia into TMA and that TMA is further converted to TMAO in humans. Moreover, we also found several genes related to antibiotic resistance, such as beta-lactamase, teicoplanin resistance genes (*tcaABR*), and mutations related to fluoroquinolones resistance.

### Accession number(s).

This whole-genome shotgun project for strain bTMA-013 has been deposited at DDBJ/ENA/GenBank under the accession number PIYR00000000. The version described in this paper is the first version, PIYR01000000.
